# Extracellular vesicles in kidney development and pediatric kidney diseases

**DOI:** 10.1007/s00467-023-06165-9

**Published:** 2023-09-29

**Authors:** Tunahan Ergunay, Federica Collino, Gaia Bianchi, Sargis Sedrakyan, Laura Perin, Benedetta Bussolati

**Affiliations:** 1 Department of Molecular Biotechnology and Health Sciences, University of Torino, Torino, Italy; 2 Laboratory of Translational Research in Paediatric Nephro-Urology, Fondazione Ca’ Granda IRCCS Ospedale Maggiore Policlinico, Milan, Italy; 3 Department of Clinical Sciences and Community Health, University of Milano, Milan, Italy; 4 Paediatric Nephrology, Dialysis and Transplant Unit, Fondazione IRCCS Ca’ Granda Ospedale Maggiore Policlinico, Milan, Italy; 5 GOFARR Laboratory, Children’s Hospital Los Angeles, Division of Urology, Saban Research Institute, Keck School of Medicine, University of Southern California, Los Angeles, CA USA; 6 Molecular Biotechnology Center, University of Turin, via Nizza 52, 10126 Turin, Italy

**Keywords:** Extracellular vesicles, Pediatric kidney diseases, Kidney development, Biomarker discovery, Therapeutic tool, Kidney transplantation, Genetic kidney diseases

## Abstract

Extracellular vesicles (EVs) are membranous cargo particles that mediate intercellular communication. They are heterogeneous in size and mechanism of release, and found in all biological fluids. Since EV content is in relation to the originating cell type and to its physiopathological conditions, EVs are under study to understand organ physiology and pathology. In addition, EV surface cargo, or corona, can be influenced by the microenvironment, leading to the concept that EV-associated molecules can represent useful biomarkers for diseases. Recent studies also focus on the use of natural, engineered, or synthetic EVs for therapeutic purposes. This review highlights the role of EVs in kidney development, pediatric kidney diseases, including inherited disorders, and kidney transplantation. Although few studies exist, they have promising results and may guide researchers in this field. Main limitations, including the influence of age on EV analyses, are also discussed.

## Introduction

Extracellular vesicles (EVs) represent an evolutionarily conserved mechanism of cell-to-cell communication in both eukaryotic and prokaryotic organisms. They are lipid membrane-covered cargo particles that contain nucleic acids, proteins, and other biomolecules, loaded through selective and/or non-selective mechanisms [1, 2]. Since the beginning of EV-related studies, EVs have been isolated from almost all mammalian cells and biological fluids, such as blood, urine, milk, and saliva [1, 2].

EVs are categorized by their characteristics such as size (small or large EVs), type of biogenesis (exosomes and ectosomes) and physiological and pathological condition of release (i.e., oncosomes, migrasomes) [3]. EVs are composed of a membrane containing surface proteins, tetraspanins and receptors, and, on the inside, cell-type specific proteins, enzymes, signal transduction molecules, chaperones and nucleic acids (e.g., microRNA, mRNA and DNA). Once released, EVs travel short distances through the extracellular matrix and long distances through biological fluids to reach the target cell [1]. More importantly, EVs present a distinct surface repertoire that allows them to target specific cell types [4]. For instance, tumor-derived EVs were reported to display a specific organotropism through integrins (α_6_β_4_ for lung and α_v_β_5_ for liver metastasis) [5].

Once internalized, EV content contributes to cellular processes such as cell metabolism, signal transduction and modulation of gene expression profile [6]. Depending on the internalization pathway and cargo content, EVs can subsequently undergo lysosomal degradation or be recycled back to the extracellular space.

**Fig. 1:**
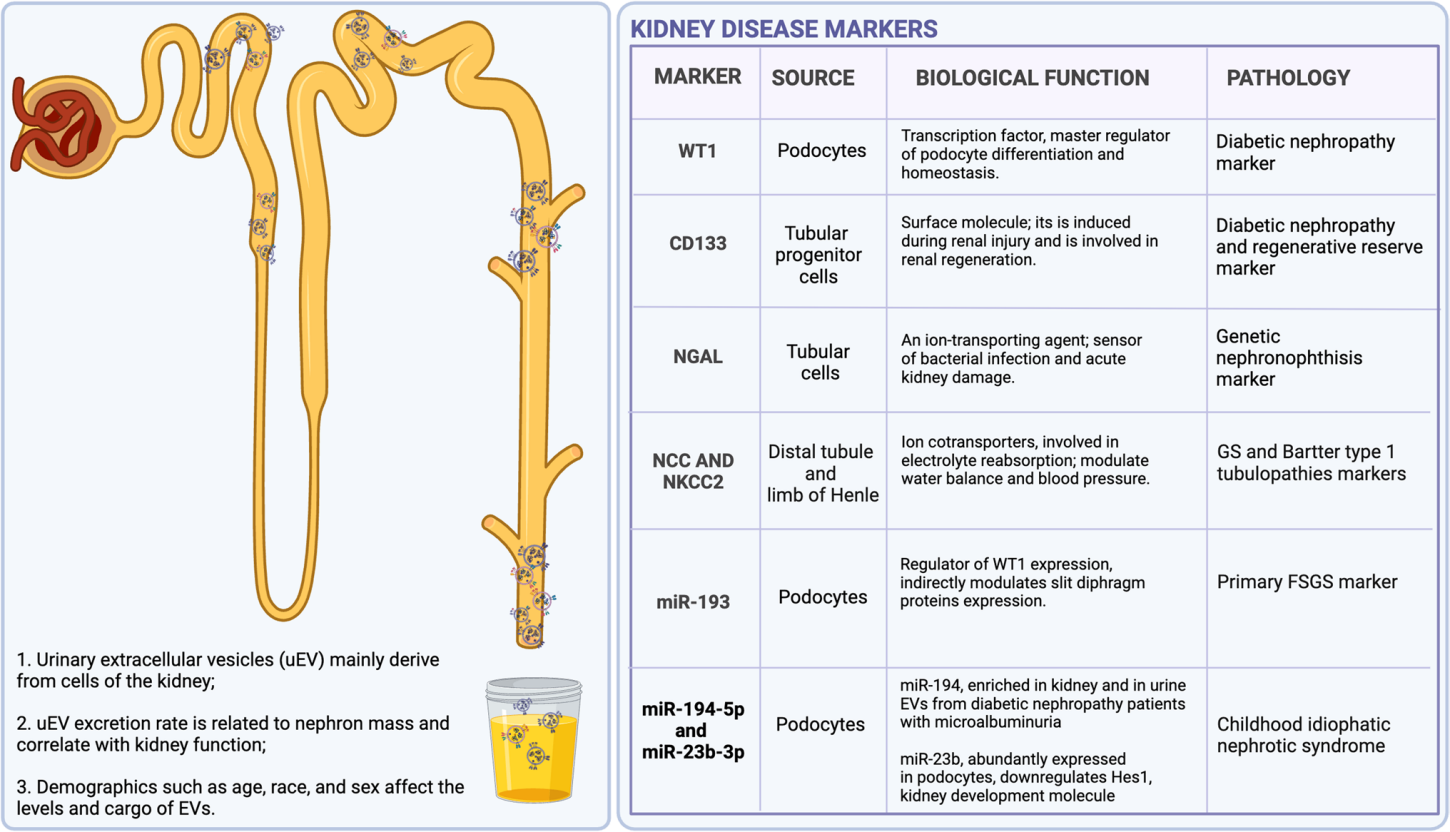
Urinary EVs are influenced by nephron mass and demographic factors and show distinctive protein cargo as disease biomarkers. Illustrations were created with Biorender.com

In general, through targeted cell reprogramming, EVs are considered highly involved in controlling organ physiology and disease modulation and progression [5–7]. In addition, increasing evidence suggests that the analysis of EV corona, the surface cargo that attaches to the EV surface through ionic bounds during their permanence in a biological fluid, might also be of interest for diagnostic applications. Urinary EVs are highly interesting for evaluating kidney physiology and pathological conditions, since they are mainly derived from the kidney (Fig. 1) [8]. In fact, serum EVs cannot pass through the membrane pores of the glomerular filtration barrier (6 nm) [9], and their presence could be a possible marker of kidney diseases. The present review highlights the potential role of EVs in kidney development, in identifying biomarkers for genetic and non-genetic primary pediatric kidney diseases, and their potential use as a therapeutic tool.

## EVs in kidney physiology and development

EVs present in urine have been involved in communication between intra-nephron compartments, including glomerular-tubular, endothelial cell-podocyte and tubular-interstitial cell interactions [10]. It is of interest to identify the factors that modulate urinary EV release and their content, and to evaluate whether EVs may regulate physiological processes within the nephron and trigger pathological events such as epithelial-mesenchymal transition.

EVs are considered to be involved in organ development. For instance, their relevance has been highlighted in neural development and regulation of stem cell numbers in epithelia such as skin and intestine [11]. The role of EVs in kidney development has also been studied. EVs isolated from a ureteric bud cell line contain proteins required for tissue morphology, homeostasis, and integrity [12]. In addition, ureteric bud-derived EVs contain miRNAs able to affect the Wnt pathway, which play an essential role in the kidney developmental process, including tissue renewal, cell proliferation and homeostasis [13]. This study underlines the important role of EV communication during nephrogenesis, and additional studies will be of interest to fully understand the EV mechanisms of action during kidney development. A recent study found that EVs from engineered kidney tubular epithelial cells improve tubuloid maturation [14]. Indeed, EVs from proximal tubule epithelial cells overexpressing organic anion transporter, a membrane transporter involved in the excretion of waste, enhanced tubuloid polarization, cilia structure formation and epithelial transport capacity. Some authors also suggest that EVs can be a major tool for developing bioartificial kidneys by promoting dense cilia structures and organoid differentiation and maturation [14].

## EV-based biomarker discoveries for pediatric kidney diseases: promises and limitations

At present, kidney biopsy, albeit invasive, represents a pivotal diagnostic tool for several pediatric kidney diseases. Diagnostic, predictive, and prognostic biomarkers are very much needed, and EVs isolated from biological fluids are under extensive investigation, as reported in Table 1. EV-based diagnostic assays may prospectively present benefits compared to the currently available ones [25]. First, the sample collection is an easy, rapid and painless process compared to traditional biopsy sampling. Second, biomarkers are protected from degradation by a lipid bilayer and can be detectable with high sensitivity as they can be concentrated in small volumes. For instance, the NGAL protein level of EVs allowed early detection of tissue injury, in patients with type 1 diabetes, in comparison with free (non EV-associated) NGAL [15]. Third, EVs provide different types of molecular diagnostic analysis based on cargo content, such as proteins, RNA species, DNA and lipids. In addition, EV-based biomarkers offer a multiplex diagnostic platform which can relate an identified biomarker to the physiopathological state of the originating cell, thanks to specific cell-related markers. In particular, origins of plasma and urinary EVs have been clearly profiled based on cell-specific signatures [26–28].

**Table 1:** Studies related to biomarker discovery in EVs isolated from pediatric patients with kidney diseases

Disease	Participants	EV origin	Biomarkers	Ref
Diabetic kidney disease	34 type I diabetes mellitus and 15 healthy controls	Urine	Presence of NGAL in patients	[15]
Acute kidney injury after cardiac surgery	14 patients and 10 controls	Blood	miR-7g-5p, miR-152-3p and miR-320a	[16]
Chronic kidney disease	27 CKD patients and 3 healthy controls	Urine	Decreased Mucin 1 protein Increased MGAM protein	[17]
Atherosclerosis and arterial stiffness in CKD	37 dialysis patients, 33 pre-dialysis patients and 18 healthy controls	Blood	Increased CD144 and CD146 protein	[18]
Acute and chronic glomerular diseases	12 Chronic and 26 Acute postinfective glomerulonephritis and 7 healthy controls	Urine	Decreased CD133	[19]
Idiopathic NS	129 Idiopathic NS patients 126 healthy controls	Urine	Increased miR-194-5p, and miR-23b-3p	[20]
Subgroups of Idiopathic NS	33 NS patients and 7 healthy controls	Urine	Different protein profile among subgroups of idiopathic NS patients	[21]
Primary FSGS	8 primary FSGS patients and 5 minimal change disease	Urine	Increased miR-193a	[22]
FSGS	20 primary nephrotic syndrome and 10 healthy controls	Urine	Increased STAT-3	[23]
Wilms tumor	14 Wilms tumor patients and 14 healthy controls	Plasma	Increased PD-L1	[24]

However, there are some limitations linked to EV studies. The heterogenic distribution of EVs in biological fluids and the absence of standards for EV isolation and purification may result in differences among studies. EV isolation requires expensive and specialized equipment like ultracentrifuge or EV-capturing kits. Moreover, the presence of contaminants in biological fluids, such as uromodulin in urine, interferes with EV isolation and analysis. In addition, the EV profile can be influenced by different physiological conditions, such as age, sex, circadian rhythm and exercise, roles that have not been fully depicted at present [29]. For instance, urinary EV expression of podocyte and mesangial cell markers was reported to decrease with age and vary according to kidney quality and sex [30]. Another study indicated that circulating miRNAs that were found significantly modulated in pediatric patients with acute kidney injury were not confirmed in infant patients, indicating the age-dependent changes in EV miRNA profile as an additional confounding factor [16].

Finally, studies often report observations in small cohorts, and large multicentric studies with consistent standardization are required to validate the present results.

## EV-based biomarkers in chronic kidney disease

The kidney is the major source of urinary EVs, whose levels directly correlate with kidney function (Fig. 1), indicating that the EV level may be a useful marker of nephron number/activity to assess chronic kidney disease (CKD) [8]. In particular, the excretion rate of urinary EVs was positively correlated with glomerular filtration rate (GFR), creatinine clearance and total kidney volume. In parallel, the number of urinary EVs significantly decreased after nephrectomy as a consequence of nephron loss.

Besides the direct number of EVs, other studies report the identification of EV-carried molecules as biomarkers. For instance, several urinary EV miRNAs involved in kidney fibrosis, such as miR-29c and miR-451, were proposed as CKD biomarkers [31, 32]. Another study investigated the urinary EV protein signatures in pediatric patients with CKD due to kidney hypoplasia or other pediatric pathologies [17]. The level of Mucin 1, a distal tubule/collecting duct-specific protein [33], was reduced and possibly correlated with nephron loss. In parallel, the proximal tubule protein MGAM, an enzyme that is involved in the digestion of starch to glucose [34], was increased, as possible compensation for the nephron loss. In another study, circulating EVs from kidney transplanted patients were analyzed to investigate kidney graft function [35]. Three miRNAs (miR-21, miR-210 and miR-4639) correlated with eGFR level and were suggested to be linked to chronic allograft dysfunction. Therefore, these studies and others indicate that EVs may be used to monitor kidney donor suitability for transplantation and allograft function. Unfortunately, no study has been performed on transplanted pediatric patients.

EV-related biomarkers for atherosclerosis and arterial stiffness were also investigated in circulating EVs from pediatric CKD patients [36], reporting that the number of EVs carrying endothelial markers, CD144 and CD146, was significantly increased compared to healthy individuals, as reported in adult patients [18]. Besides, the levels of CD144^+^ and CD146^+^ EVs were positively associated with blood pressure, age and C-reactive protein level and negatively associated with hemoglobin, eGFR and albumin level. Therefore, CD144^+^ and CD146^+^ EVs in circulating blood may be potential biomarkers for atherosclerosis and arterial stiffness in pediatric CKD patients.

## EV-based biomarkers in acute glomerular injury

Urinary EVs also appear as a potential biomarker of glomerular injury. For instance, urinary EV level of podoplanin and Wilms tumor 1 (WT1) protein are considered as potential markers of diabetic glomerular damage and podocyte injury, respectively [37, 38].

In a recent study, urinary EV expression of CD133, a marker of renal progenitors, was shown to be decreased in children with acute glomerulonephritis during the phase of kidney damage, but the level could be restored with patient treatment and recovery [19]. Another study on adult transplanted patients, CD133 levels were shown not only to correlate with glomerular filtration rate, but also to predict the kidney progression toward CKD, suggesting that CD133 levels might be related to kidney regenerative capacity after damage [28].

A pioneering proteomic analysis [39] on urinary EVs isolated from young adults affected by either IgA nephropathy or thin basement membrane nephropathy (TBMN), allowed the identification of 1877 exosomal proteins that are differentially expressed in patients compared to healthy controls. Among these, the expression levels of four proteins (aminopeptidases N, vasorin precursor, α-1-antitrypsin, and ceruplasmin) were confirmed by Western Blot. The analysis of these factors not only allows diseased individuals to be separated from healthy ones, but also distinguishes children affected by IgA nephropathy from those with TBMN. This finding is remarkable as the initial clinical features of the two groups are mostly overlapping, but the clinical evolution is significantly different, with TBMN having a favorable prognosis for most patients, while IgA nephropathy leads to kidney failure in 15–40% of cases [40].

## EV-based biomarkers in nephrotic syndrome

Nephrotic syndrome (NS) is the most common pediatric glomerular disease, with a worldwide incidence of 5 in 100,000 children per year [41]. The first-line therapy for NS is treatment with corticosteroids (i.e., prednisone), but 10% of patients are non-responsive [42]. Responsiveness to the treatment is currently the main criteria for patient classification, so that they are defined as steroid-sensitive or steroid-resistant. However, the stratification of these patients still lacks details and, especially for those with steroid-resistant NS, this leads to unmet therapeutic needs. For these reasons, EV-based markers could be relevant for classifying subtypes, etiologies or steroid treatment response. Due to technical challenges in urinary EV isolation and characterization associated with the nephrotic range of proteinuria, only a few studies have been performed on urinary EVs in NS patients, mainly focusing on their miRNA and protein content.

The miRNA profile of urinary EVs was assessed in a large cohort of pediatric patients with NS [20]. The authors found that 30 miRNAs were significantly increased in urinary EVs of NS patients. In particular, 5 of these (miR-194-5p, miR-146b-5p, miR-378a-3p, miR-23b-3p and miR-30a-5p) were demonstrated to be more than 3 times higher in active NS than in patients with clinical remission. Among those miRNAs, miR-194-5p and miR-23b-3p levels correlated with urine protein levels, suggesting that these miRNAs could represent a promising diagnostic biomarker in childhood idiopathic NS. When studying NS patients classified according to their steroid treatment response, EV protein patterns were found to differ among patients with different subgroups and specifically correlated with patient response [21]. Based on the first study, urinary EV markers might be useful as prognostic factors in idiopathic NS.

WT1 protein was identified as a relevant marker for podocyte-related diseases in a variety of studies [43]. In one study, a role for WT1 protein in focal segmental glomerulosclerosis (FSGS) pathogenesis was proposed by Gebeshuber et al., who demonstrated that increased levels of miRNA-193a inhibit the expression of WT1, leading to a decreased expression of some relevant architectural podocyte proteins [22]. However, contradictory results were published in two studies related to the relevance of EV-expressed WT1 to steroid responsiveness. Zhou et al. showed that urinary WT1 discerns FSGS patients from steroid-sensitive ones with active pathology and allows us to distinguish steroid-sensitive patients in remission from those in relapse [38]. On the other hand, urinary exosomal WT1 did not correlate with either steroid responsiveness, or the kidney pathological condition according to Lee et al. [44]. These differences between results may be caused by the age-related changes of WT1 in urinary EVs. Another recent study focused on STAT3 activation in patients suffering from FSGS [23]. Urinary EVs from pediatric patients with FSGS promoted STAT3 phosphorylation and mesangial cell proliferation, suggesting their role in mesangial cell proliferation through STAT3 pathway activation.

## EV-based biomarkers in genetic kidney diseases

Gitelman syndrome (GS) and Bartter type 1–4 syndromes are rare inherited forms of hypokalemic metabolic alkalosis [45, 46]. Currently, their differential diagnosis is performed mainly by clinical evaluation and genetic testing [47]. A pilot study from Corbetta et al. [48] demonstrated the usefulness of urinary EV measurement of NCC and NKCC2, ion-transporter proteins in the distal tubule and limb of Henle, respectively, in distinguishing GS and Bartter type 1 patients from healthy controls and from other salt-losing tubulopathies. In detail, urinary EVs from GS patients were characterized by a lower level of NCC compared to healthy controls, while lower NKCC2 levels allow to discriminate Bartter type 1 subjects from other patients. For future studies, the authors suggested investigating the correlation between genetic mutation penetrance and ion-channel protein levels in urinary EVs, because the NCC and NKCC2 expression levels could also be related to the mutation severity of the genetic variation. Urinary EV characterization has also been proven to increase the power of diagnosis of the familial form of nephronophthisis, an autosomal recessive kidney ciliopathy with high genetic variability and a strong phenotypical heterogeneity [49]. Urinary EV biomarker analysis in pediatric patients is still in its infancy, with a single study performed in 2019 by Stokman et al. [50], where candidate urinary EV biomarkers were identified through global proteomic analysis. Among 156 urinary EV proteins identified as differentially expressed by mass spectrometry in 12 pediatric patients and controls, the upregulation of vesicle NGAL was observed in the patient cohort, corroborating with its enrichment in serum and urine of nephronophthisis subjects [51]. Thus, the increase in NGAL protein level in urinary EVs provides an early detection for organ damage in nephronophthisis patients.

In general, urinary EV assessments might be of interest in genetic diseases as they could represent an early test in the first disease stages before performing the genetic evaluation or they could be useful in the stratification of the genetic disease based on mutation penetrance, allowing for a better characterization of the severity of the disease and therefore better management. However, at present, there is no indication that EV studies might have better diagnostic precision over genetic testing.

## Wilms tumor

Among kidney diseases, Wilms tumor is the most common pediatric kidney malignancy and recurrence may occur in about half of pediatric patients [52, 53]. EV-related prognostic markers are extensively studied in oncology. In Wilms tumor, however, a single study focused on the analysis of EV-expressed immune checkpoint molecule PD-L1 as a Wilms tumor progression marker. When evaluating PD-L1 levels on plasma EVs from fourteen Wilms tumor patients [24], PD-L1 appeared significantly correlated with CD8^+^ T cell function inhibition. However, the study has weak spots; for example, no change was observed in some of the effector T cell markers when T cells were co-cultured with PD-L1 EVs of patients, and no correlation between those markers and PD-L1 was observed. Further studies are needed to examine the relationship between PD-L1 EVs and CD8^+^ effector T cell activation in pediatric patients with Wilms tumor.

## Therapeutic use of EVs for pediatric kidney diseases

In addition to biomarker discoveries, EVs are a promising tool for new therapeutic approaches [54]. For instance, administration of mesenchymal stem cell (MSC)-derived EVs induced reduction of kidney inflammation, prevention of kidney failure, and decreased kidney fibrosis in several in vitro and in vivo models [55]. In particular, EVs have been mainly studied in models of acute kidney injury with tubular damage, or of CKD with kidney fibrosis, whereas few studies investigated their specific effect on glomerular injury. We recently showed the beneficial effect of MSC-EVs on podocyte damage in a millifluidic model of glomerular filtration barrier in vitro [56]. Likewise, endothelial progenitor cell-derived EVs protected podocytes from apoptosis and prevented nephrin shedding induced by complement damage [57].

The utility of exogenous EV therapy in a progressive CKD setting is a new area of investigation. Recently, the renoprotective effect of amniotic fluid stem cell (AFSC)-derived EVs on glomerular endothelial injury in Alport syndrome [58], a progressive CKD characterized by ColIagen IV mutation leading to kidney failure was demonstrated [59]. A single administration of stem cell derived-EVs before onset of heavy proteinuria was able to prevent serum creatinine and albuminuria value increases in an X-linked mouse model of Alport syndrome, resulting in significantly improved kidney function up to 28 weeks post-treatment relative to non-treated controls. This effect was similar to that observed by administration of the parent cells, AFSC [60]. In vitro, AFSC-EVs, due to the high abundance of their surface VEGFR1 expression, functioned as a trap for excess VEGF, preventing downstream activation of the canonical VEGF/VEGFR2 signaling in glomerular endothelial cells. This indicates that modulation of VEGF within the glomeruli of Alport mice (highly elevated in the early stage of disease) may involve EV-dependent trapping of VEGF as one possible mechanism of action. In addition, AFSC-EVs contain a high abundance of other angiomodulatory miRNA cargo (miR-16.1, miR-93, miR23a, miR-27a, miR-221, miR-322 and miR-145), which may have further contributing roles, and some of which are already under investigation [58].

## EVs in the clinic

Further, a clinical trial was performed on patients with CKD [61]. In this phase II/III study, 40 patients with CKD stage III or IV were treated with two doses of umbilical cord MSC-EVs. The EV-treated group showed significant improvement in overall kidney function. Therefore, this clinical study strengthens the idea of the therapeutic use of EVs in kidney diseases and encourages other researchers to pay attention to this promising field for both adult and pediatric patients. Therapeutic EVs can also be engineered or combined with synthetic vesicles (EV hybrids) depending on the purpose, cargo content, target specificity and delivery method [62].

## EVs in kidney transplantation

Some studies suggest that EVs have essential roles in immunization within kidney transplantation [63]. Although no clinical study has been conducted regarding the therapeutic use of EVs as a suppressor for allograft rejection, a paper recently collected and meta-analyzed the findings of preclinical models to investigate the beneficial effects of EVs in transplantation [64]. This analytical review of seven preclinical studies investigated immune cell and MSC-EVs from syngeneic and allogeneic models regarding graft survival and kidney function. Although no beneficial effect was observed using MSC-EVs, syngeneic and allogeneic immune cell-derived EVs enhanced graft survival, suggesting the therapeutic use of EVs as a suppressor of allograft rejection.

## Conclusions

Kidney diseases are one of the fields of interest for EV researchers. Many preclinical and clinical studies focus on EV roles in different kidney diseases. Because urine is easy to collect, urinary EVs become an opportunity to find biomarkers of diseases. However, several challenges need to be solved for standardization and reproducibility of EV studies [65]. Especially for pediatric disease studies, the urinary EV analysis might also be impacted by age differences with respect to the adult population. We found a limited number of pediatric studies, primarily for biomarker discoveries, whereas no study related to therapeutic use of EVs in pediatric kidney disorders or transplantation is present. We strongly recommend that researchers investigate EVs in pediatric kidney diseases for diagnosis and therapy.

## Abbreviations

CKD: Chronic kidney disease
EV: Extracellular vesicles
FSGS: Focal segmental glomerulosclerosis
GFR: Glomerular filtration rate
GS: Gitelman syndrome
miRNA/miR: Micro-RNA
MSC: Mesenchymal stem cell
NS: Nephrotic syndrome
PDE: Peritoneal dialysis effluent
TBMN: Thin basement membrane nephropathy
